# Lectures on Auto-Intoxication in Disease

**Published:** 1894-10

**Authors:** 


Lectures on Auto-Intoxication in Disease or Self-poisoning of the Individual. By Ch. Bouchard. Translated with a preface by Thomas Oliver, M. A., M. D., F. R. C. P. Philadelphia, Penna.: The F. A. Davis Company, London: F. J. Rebman, 1894.

This learned book deserves an extended review and indeed a much earlier notice than we have given it. But there is so much in it, and it requires such close thought, and the time at the disposal of the editor is so limited that a considerable period of time has elapsed since it was first brought to our notice.

As the title-page explains it is an inquiry into the phenomena of the human system by its own fluids and the products of the processes going on within it to maintain its physiological life.

As the author well says (page 9), "that the physician ought not to allow himself to be absorbed alone in research after a microbe. He ought to occupy himself with the infectious agent; but he ought to retain a good deal of his anxiety for the study and research of circumstances which disarm the organism against the invasion of that agent. When the physician shall be in possession of this double knowledge that many diseases are produced by microbes, and that these can act only by means of deterioration of the health resulting from various pathogenetic processes, he will recognize that the new discoveries contain nothing subversive, and that the lessons taught by ancient medical observations are not compromised."

In directing the reader's attention to the sources of poisoning in the individual, the author says: "In the first rank are placed the mineral substances introduced with our food; then come the products of physiological secretion—saliva and bile; the products of digestion; digestion, too, while it transforms albuminoid substances into peptones, also gives birth to alkaloidal poisons; and, lastly, toxic substances resulting from intestinal putrefactions."

The whole scheme of this fine book is a discussion of these sources of poisoning and an explanation of their mechanism of action. All this is much too elaborate to be extensively quoted here, and the best plan is to get the book and read it. It is specially advisable to read the work for one's self, as it deals with subjects of every-day interest to the practitioner, and gives him the key to the explanation of many a case of death where the most searching examination fails to discover any actual lesion that would account for it.

